# Supplementary material for: How does the farmland management rights mortgage loan affect food security: Based on the evidence of major grain producing areas in China
Source: PLoS One. 2025 Apr 28;20(4):e0313881. doi: 10.1371/journal.pone.0313881 (PMC12036842; doi:10.1371/journal.pone.0313881)
Supplement: Attachment — Submitted filename: comments_plos0916.docx [file pone.0313881.s002.docx]

This study examines the impact of farmland management rights mortgage loans (FMRML) on food security in China's major grain-producing areas. Using panel data from 1234 counties from 2010 to 2021 and employing a Difference-in-Differences model, the research finds that the FMRML policy significantly enhances food security. The following are my comments on the abstract of this paper.

Major：

1. While the introduction provides background on food security and the FMRML policy in China, it doesn't clearly articulate the specific gap in the existing literature that this study aims to fill. The authors should more explicitly state what is currently unknown or understudied regarding the relationship between FMRML and food security, and how their research contributes new knowledge to this area. This would help justify the importance and novelty of the study.
2. While the introduction mentions that the study will "theoretically analyze" the impact of FMRML on food security, it doesn't provide a clear theoretical framework or hypotheses. The authors should outline the theoretical mechanisms through which they expect FMRML to affect food security, and present specific hypotheses that will be tested in the empirical analysis. This would provide a stronger foundation for the subsequent empirical work.
3. While the study considers regional differences, the analysis of policy effect heterogeneity remains superficial. I recommend further exploration of the differentiated impacts of the FMRML policy across various farmer types (e.g., smallholders vs. large-scale operators), different agricultural management models (e.g., grain cultivation vs. cash crops), and diverse land tenure structures. This would provide more targeted evidence for precise policy implementation and reveal the complex interactions between land mortgage loan policies and agricultural production structures, as well as rural social structures.
4. The conclusion should explicitly acknowledge the limitations of the current study and suggest directions for future research. This might include discussing data limitations, potential omitted variables, or alternative methodological approaches that could further validate or extend the findings. Specific areas for future investigation could be proposed, such as long-term effects of FMRML on agricultural productivity, impacts on different crop types, or effects on rural household welfare beyond food security.

Minor：

1. Provide a more comprehensive and critical review of relevant literature. Clearly identify gaps in existing research and explicitly state how your study addresses these gaps. Synthesize previous findings rather than merely summarizing individual studies.
2. In the discussion section, go beyond simply restating results. Interpret your findings in the context of existing literature and broader theoretical frameworks.
